# Concurrent Validity and Reliability Between Noraxon Ultium™ IMU and Vicon OMC for Lower Limb Gait Assessment Across Variable Walking Speeds

**DOI:** 10.3390/s26144520

**Published:** 2026-07-16

**Authors:** Trung T. Le, Ha V. Vo, Scott C. E. Brandon

**Affiliations:** 1 Department of Biomedical Engineering, School of Engineering, Mercer University, Macon, GA 31207, USA; vo_hv@mercer.edu; 2 Department of Interdisciplinary Engineering, College of Engineering, University of Guelph, Guelph, ON N1G 2W1, Canada; scott.brandon@uoguelph.ca; 3 Department of Orthopedic Surgery, School of Medicine, Mercer University, Macon, GA 31207, USA

**Keywords:** IMoCAP, OMC, gait analysis, validation, lower-limb kinematics, Bland–Atman agreement, reliability, RMSE, walking speed

## Abstract

**Highlights:**

**What are the main findings?**

- The Noraxon Ultium™ IMU demonstrated moderate agreement with optical motion capture for sagittal-plane kinematics, with low RMSE and moderate reliability across walking speeds in healthy individuals.
- Frontal-plane measures showed moderate agreement, while transverse-plane kinematics exhibited the largest errors and lowest reliability, particularly at higher walking speeds.

**What are the implications of the main findings?**

- The Noraxon Ultium™ IMU systems can be used for monitoring joint angles in the sagittal-plane gait assessment and supporting field-based applications.
- Caution is required for multi-planar kinematics, as accuracy is influenced by walking speed, calibration quality, and modeling assumptions.

**Abstract:**

**Background:** Wearable inertial measurement units (IMoCAPs) are increasingly used in clinical gait analysis due to their portability and ability to capture data outside laboratory settings; however, validation across operating conditions is essential. **Objective:** To evaluate the concurrent validity and reliability of the Noraxon Ultium™ IMU system against a Vicon optical motion capture (OMC) system for lower-limb kinematics during walking across different speeds and time intervals. **Methods:** Ten healthy adults performed overground walking at slow, normal, and fast self-selected speeds. Kinematics were recorded simultaneously using both systems. Discrete variables (Max, Min, ROM) were analyzed using three-factor repeated-measures ANOVA (Device × Speed × Time, *p* < 0.05). Agreement was assessed using Bland–Altman analysis, RMSE, and ICC (3,1), and waveform differences were evaluated using Statistical Parametric Mapping (SPM). **Results:** Time effects were minimal across all planes. Sagittal-plane kinematics showed strong agreement, with small biases (<3°), low RMSE (≤2.5°), and moderate reliability (ICC = 0.65–0.74). Both systems detected increased hip and knee motion with speed, although Device × Speed interactions indicated greater IMoCAP underestimation at higher speeds. Frontal-plane agreement was poor to moderate (RMSE: 1–3°, ICC: 0.42–0.74). Transverse-plane kinematics demonstrated the largest discrepancies (RMSE up to 6–7°, ICC: 0.17–0.26), particularly for hip rotation. SPM revealed significant waveform differences across all planes. **Conclusions:** The Noraxon Ultium™ IMU provides valid sagittal-plane gait assessment, moderate frontal-plane agreement, and limited reliability for transverse-plane kinematics, requiring cautious interpretation at higher speeds.

## 1. Introduction

Understanding human posture and movement is essential in clinical rehabilitation, as it elucidates deviations between normal and pathological patterns [1,2]. Quantifying symmetry and asymmetry during motion can signal progressive improvement, facilitate early intervention, and guide patients toward functional milestones, ultimately aiming to restore their mobility [1,3,4,5]. In individuals with lower-limb amputation, for example, gait analysis is particularly valuable for evaluating prosthetic alignment, socket fit, and component performance [1,5]. As rehabilitation shifts toward evidence-based and personalized care, reliable motion capture technologies have become indispensable in both research and clinical practice [1,2]. Currently, qualitative and quantitative movement assessment has relied on gait analysis techniques.

The current gold standard is marker-based motion capture (OMC), which uses infrared cameras to track passive reflective markers placed on anatomical landmarks with sub-millimeter accuracy [6,7,8,9]. This approach enables precise reconstruction of joint angles and segmental kinematics, delivering high spatial and temporal accuracy validated across research and clinical contexts. However, OMC is constrained by high cost, lack of portability, the need for trained operators, extensive setup, calibration, and post-processing, and confinement to dedicated laboratory settings [7,10,11]. These limitations severely restrict its utility in real-world or field-based clinical environments.

In contrast, inertial motion capture (IMoCAP) systems leverage wireless, wearable inertial measurement units (IMUs), which integrate accelerometers, gyroscopes, and magnetometers to estimate segment orientation and compute joint angles via sensor fusion algorithms. IMU orientation is tracked by means of a quaternion-based time integration of the angular velocity vector as measured by the gyroscope and corrected by comparing the resultant orientation versus the inclination estimated from the accelerometer and heading estimated from the magnetometer [6,11,12,13,14,15]. Compared with optical OMC, IMoCAP eliminates the need for external cameras or markers, significantly reducing setup time and improving accessibility for clinicians and patients. Its portability and cost-effectiveness enable gait assessment in natural environments and supports continuous monitoring of functional mobility during daily activities. Nevertheless, IMoCAP remains susceptible to sensor drift, magnetic interference, and soft tissue artifacts, which can compromise accuracy relative to optical systems [16]. Unlike OMC, which directly measures marker positions, IMU-based systems depend on algorithmic estimation and sensor fusion, potentially introducing systematic or random errors depending on calibration methods and activity type [2,12].

Accurate quantification of gait kinematics using wearable IMoCAP systems such as the Noraxon Ultium is increasingly important for clinical populations, including individuals with cerebral palsy, osteoarthritis, stroke, and lower-limb amputation, where movement patterns are often variable. While prior validation studies have demonstrated acceptable agreement between IMU and OMC systems under controlled conditions, these evaluations are typically conducted at self-selected or fixed walking speeds and over short recording durations in healthy individuals [7,9,10,17]. However, both walking speed and time-dependent factors (e.g., sensor drift and soft tissue artifact) are known to influence kinematic outputs and may introduce systematic error in IMU-based measurements [18]. Speed variations alter joint excursions, segment accelerations, and dynamic alignment, potentially challenging sensor fusion algorithms and model assumptions, while prolonged data collection may exacerbate orientation drift and calibration sensitivity, particularly in field-based environments [11]. These effects are likely amplified in clinical populations, where asymmetry, compensatory strategies, and inconsistent gait patterns further challenge model robustness. The Noraxon Ultium™ IMU with an on-board calibration allows real-time model correction referencing with the subject’s walking calibration and this feature was one of the pivotal advancements available in the recent update to Noraxon’s Myoresearch (MR 3.18) software. Investigating the interaction between walking speed and time is critical to determine whether the Noraxon Ultium™ IMU system can reliably capture kinematic changes across realistic and clinically relevant conditions. Establishing this validity is essential to support its clinical application in longitudinal monitoring and intervention assessment for populations with neuromuscular and musculoskeletal impairments [19].

Before the Noraxon Ultium™ IMU system gait analysis can be translated into pathological populations, validation under controlled healthy gait conditions remains necessary to establish baseline system performance and identify methodological limitations. Therefore, this study aimed to concurrently evaluate the validity and reliability of the Noraxon Ultium™ IMU system against a gold-standard Vicon optical motion capture (Vicon OMC) system for lower-limb joint kinematics. Specifically, it investigated (1) the effect of walking speed, and (2) the effect of time to assess system reliability in relation to potential sensor drift. The findings will inform the feasibility of deploying IMU-based gait analysis in resource-constrained clinical settings where optical systems are unavailable. It was hypothesized that the Noraxon Ultium™ IMU system would exhibit strong agreement with Vicon OMC in sagittal-plane kinematics of the pelvis, hip, knee, and ankle, with reduced accuracy expected in the frontal and transverse planes. Additionally, it was anticipated that the system would maintain stable joint angle measurements across walking speeds, with only minimal drift-related variation over the testing duration, supporting its viability as a reliable and practical tool for clinical gait analysis.

## 2. Materials and Methods

### 2.1. Participants

Ten participants were recruited for this study, including five males (age: 29 ± 6 yrs, height: 179.3 ± 9.3 cm, weight: 81.24 ± 13 kg) and five females (age: 25.6 ± 1.1 yrs, height: 174.3 ± 7.5 cm, weight: 67.3 ± 10.6 kg). Inclusion criteria for participants were: (i) healthy individuals, (ii) between 18–65 years old, and (iii) physically able to perform walking and jogging exercises as instructed by the researcher during experiments. Experimental procedures and gait analysis were approved by the institutional Research Ethics Committee (Guelph REB: NPES 22-10-021). Participants provided written, informed consent prior to conducting the experiment.

The sample size for this study was selected based on prior validation work focusing on measurement agreement rather than population inference. In these validation studies, the primary objective is to characterize systematic bias, variability, and reliability between measurement systems under controlled conditions [7,9,17]. Berner et al. recommended that relatively small sample sizes (9–15 participants) are sufficient to achieve stable estimates of agreement metrics such as RMSE and Bland–Altman limits of agreement, provided that multiple gait cycles and repeated conditions are analyzed within each participant [9]. This approach leverages the high within-subject repeatability of gait kinematics and the large number of observations generated per participant, which together increases statistical power for system-level comparisons. Furthermore, the present sample size is consistent with established validation literature (Nijmeijer et al., 2023, N = 10 [7] and Berner et al.,2020 N = 10 [9]) and is appropriate for assessing the concurrent validity and reliability of IMU-derived gait kinematics across walking speeds and time points.

### 2.2. Instrumentation: Vicon OMC and Noraxon Ultium™ IMU

Three-dimensional gait data were simultaneously collected using two motion analysis systems: an optical motion capture (OMC) system (Vicon, Oxford, UK) and a wearable inertial measurement unit (IMU) system (Noraxon Ultium™ Portable Lab, Noraxon Inc., Scottsdale, AZ, USA). Both systems were applied concurrently to each participant to enable direct comparison of kinematic outputs.

**Figure 1:**
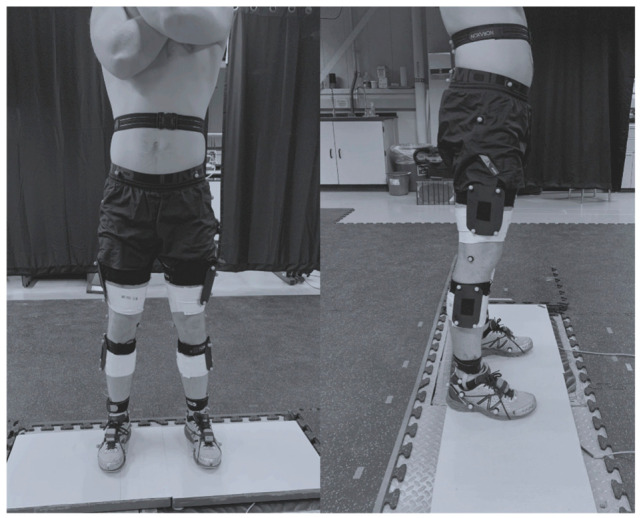
Placement of reflective markers for Vicon OMC and IMU sensors for Noraxon Ultium™ IMoCAP. Marker placement included the pelvis landmarks (anterior and posterior superior iliac spines), bilateral thigh and shank segment clusters, knee landmarks (medial and lateral femoral condyles), ankle landmarks (medial and lateral malleoli), and feet (calcaneus, first and fifth metatarsal heads). Eight wireless Noraxon Ultium™ IMU sensors were affixed to the lower thoracic region, pelvis, bilateral thighs, shanks, and feet in accordance with the Noraxon placement guidelines. The sacral IMU sensor was mounted together with the cluster of passive reflective markers to support cross-system alignment.

**Figure 2:**
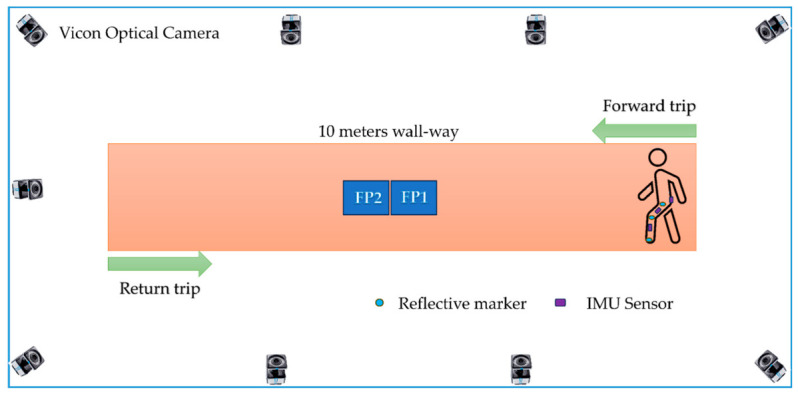
A laboratory setup for the concurrent validation between Vicon OMC and Noraxon Ultium™ IMU. Subject wearing both reflective markers and IMU sensors for concurrent recording.

For the OMC system, 38 retro-reflective markers were placed bilaterally on anatomical landmarks and tracking clusters attached to the lower extremities according to published marker set guidelines (HAS-Motion, 2024; see Figure 1). All markers were placed by a single rater trained in motion capture procedures to minimize placement bias [20]. For each participant, markers remained in place throughout all trials to ensure consistency across recordings. Marker trajectories were captured using nine (seven Bonita, two Vero) optical cameras operating at 200 Hz (see Figure 2). Raw marker data were processed with a Woltring filter (maximum gap = 5) before kinematic reconstruction.

For the IMU system, eight sensors were secured using fixation straps at the pelvis, elastic Velcro straps on the thighs and shanks, double-sided tape at the sacrum, and custom Noraxon rubber bands at the feet [10]. IMU signals were also sampled at 200 Hz and hardware-synchronized with the Vicon system at the start of each trial via a Vicon-generated voltage pulse. Raw IMU data were processed using a proprietary sensor fusion (Kalman filter), and joint kinematics were derived using the IMU-based body model implemented in MyoResearch software (MyoResearch MR 3.18) [18,19].

### 2.3. Ultium Experimental Design

Concurrent validity of the OMC and IMU systems was assessed across walking speed and time factors. Participants walked along a 10 m walkway including forward and return trips (see Figure 2) under three conditions: self-selected such as (1) comfortable normal speed, (2) slow speed, and (3) fast speed, each defined relative to the participant’s normal daily gait. The experimental rater confirmed the classification of self-selected walking speed (slow, normal, fast) with each participant prior to recording to ensure consistency. To familiarize participants with the equipment, 2–3 practice trials were performed while instrumented with both IMU sensors and reflective markers. During testing, recorded trials were collected for each of the three speed conditions (slow, normal, fast) at three measurement intervals (0, 5, and 10 min). This experimental design yielded a total of nine walking trials per participant. The order of speed conditions was randomized by each participant to minimize systematic bias. Between trials, participants rested in a seated position to reduce fatigue and prevent displacement or movement of sensors or markers. The study rater visually checked all reflective markers and IMU sensors prior to the next recording. All trials were conducted under identical laboratory conditions.

### 2.4. Kinematic Modelling

#### 2.4.1. Vicon OMC

A generic OpenSim musculoskeletal model (UMocoD v2) was used for subject specific scaling and kinematic calibration [21]. This model is based on the Rajagopal full-body model, which has 31 degrees of freedom and 84 muscles in its lower limbs and lumbar joint. This model was selected to facilitate optimal control simulations (MOCO in future work).

Model scaling was performed in OpenSim. Briefly, segment lengths were scaled to match lower-limb joint centers and upper-limb anatomical landmarks between experimental data (OMC) and the model. Then, the model’s pose and marker locations were optimally adjusted to minimize error between model-fixed and OMC marker locations during a static calibration (quiet standing) trial. Harrington’s regression equations were used to define hip joint centers, while knee and ankle joint centers were defined at the midpoint between epicondylar and malleolar markers, respectively [22,23]. Joint angles were computed using the Inverse Kinematics tool in OpenSim to minimize error between model-fixed and OMC marker positions.

#### 2.4.2. Noraxon Ultium™ IMU

Segment-specific scaling of the pelvis, lumbar region, and lower extremities was performed using landmark reference measurements in combination with anthropometric ratios derived from the Dempster model [24]. In the Noraxon Ultium™ IMU-based body virtual model (MR 3.18), subject-specific scaling parameters were computed based on the subject’s overall height as follows: pelvis width was estimated from the inter-ASIS distance (0.165*height for males, 0.185*height for females), lumbar length from C7 to the posterior superior iliac spine (0.338*height), thigh length from the lateral greater trochanter to the lateral femoral epicondyle (0.245*height), shank length from the lateral femoral epicondyle to the lateral malleolus (0.246*height), and foot length from the posterior calcaneus to the tip of the first metatarsal (0.152*height for males, 0.120*height for females). The kinematic model has six degrees of freedom (DOF) for Hip (flex/extend, abduct/adduct and internal/external rotation), Knee (flex/extend, abduct/adduct, internal/external rotation), and Ankle (dorsiflexion/plantarflexion, inversion/eversion, and abduction/adduction) joints.

For the Noraxon Ultium™ IMU system, a functional walking calibration was performed in MR 3.18. This procedure used accelerometer-based alignment to reduce sensor misorientation and correct for magnetic distortion prior to data collection. A Kalman filter was applied for sensor fusion, and real-time stabilization and correction modes were enabled to minimize drift and stabilize orientation estimates [18]. Prior to recording, each subject was asked to perform functional walking calibration including three pre-defined modes such as pre-static capture, dynamic walking, and post-static capture. During experimental recording, model calibration was directly scaled and applied to the subject. Sensor drift sometimes happens after 5 min of experimental setup, causing model distortion; this can be adjusted by applying functional calibration in real-time. Finally, three-dimensional lower-limb joint angles (pelvis, hip, knee, and ankle) were computed in MyoResearch 3.18 using a proprietary model that complies with ISB recommendations [10,25].

### 2.5. Data Processing

**Figure 3:**
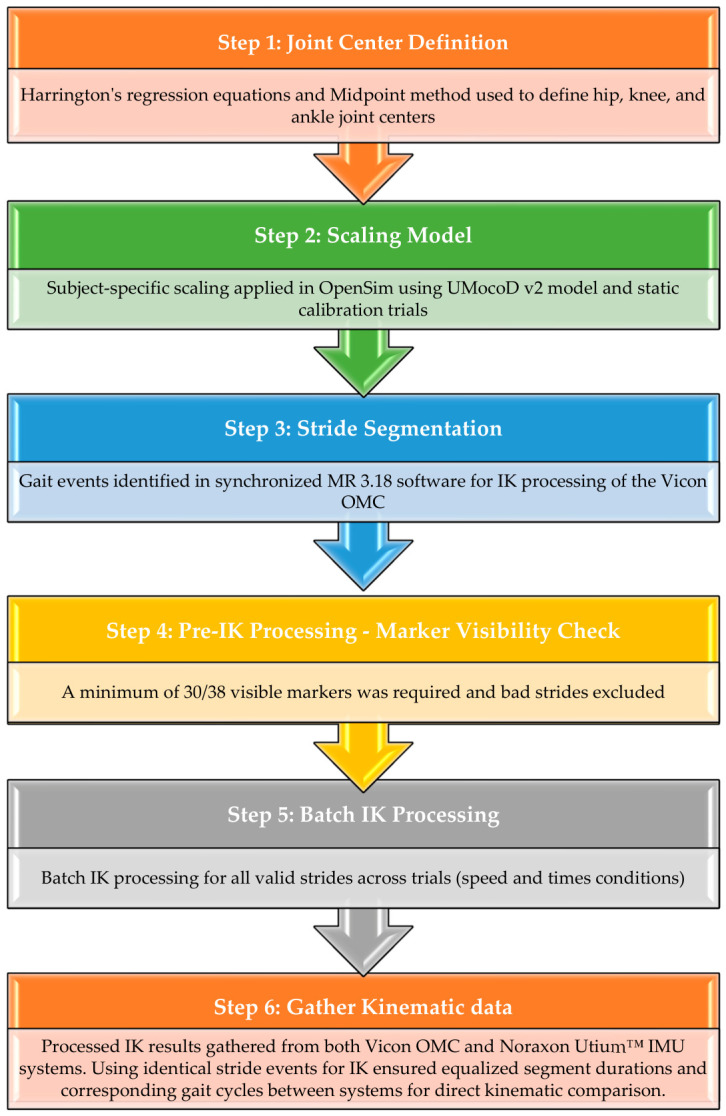
Pipeline of kinematic processing for the Vicon OMC using OpenSim API platform. All data processing was done using MATLAB (R2021a, MathWorks, Natick, MA, USA).

A custom MATLAB (R2021a, MathWorks, Natick, MA, USA) pipeline was developed to process experimental data from the Vicon OMC (using the OpenSim API), and IMoCAP systems (see Figure 3). To ensure consistency, data were segmented into strides for both OMC and IMoCAP systems using a common set of gait events defined within the IMoCAP (MR 3.18) software. Gait cycles were visually inspected during preprocessing to confirm consistency of event segmentation across systems.

### 2.6. Statistical Analysis

For each stride, the local maximum (Max), minimum (Min), and range of motion (ROM) (degrees) for each joint angle (3D pelvis, 3D hip, knee flexion, and ankle plantarflexion angles) were computed. For each speed (slow, normal, fast) and time (0, 5, 10 min), kinematic data were averaged across all strides to compute within-subject-means.

A three-factor repeated-measures ANOVA was conducted to evaluate differences between motion capture systems (Vicon OMC vs. Noraxon Ultium™ IMU) across walking speeds (slow, normal, and fast) and times (0, 5, and 10 min). Assumptions of normality and sphericity were verified using Shapiro–Wilk and Mauchly’s tests, respectively. When sphericity assumptions were violated, Greenhouse–Geisser corrections were applied. Post-hoc pairwise comparisons with Bonferroni–Holm adjustments were used to identify significant differences across conditions. Statistical significance was set at *p* < 0.05 [26]. All analyses were performed using JASP statistical software (version 0.95.4; University of Amsterdam, Amsterdam, The Netherlands) [27].

To characterize absolute agreement between systems, Bland–Altman analyses were performed Ultium for discrete joint kinematic variables (Max, Min, and ROM). For each variable, the mean difference (bias), the 95% limits of agreement (LoA = bias ± 1.96 SD), and 95% Confidence Interval (CI) were calculated. The 95% CI for the upper and lower limits indicates the precision of the LoA. A narrow CI means the LoA are estimated precisely; a wide CI means low precision.

Absolute agreement between the Noraxon Ultium™ IMU and Vicon OMC systems was further assessed using intraclass correlation (ICC). Specifically, a two-way mixed-effects, single-measure intraclass correlation coefficient ICC(3,1, absolute agreement) model evaluating absolute agreement was used. This approach was selected because the two measurement systems were considered to represent fixed methods of interest measured under identical testing conditions. ICC was examined for Max, Min, and ROM joint angles across the sagittal, frontal, and transverse planes. ICC values were interpreted using established criteria from Koo et al.’s study, where <0.50 indicates poor reliability, 0.50–0.75 moderate reliability, 0.75–0.90 good reliability, and >0.90 excellent reliability [21].

To provide an approximate, summary metric of agreement between the Noraxon Ultium™ IMU and Vicon OMC systems, root mean square error (RMSE) was calculated Ultium for each joint, plane of motion, and discrete variable (Max, Min, and ROM) across all combinations of walking speed and time. RMSE values were calculated from condition-averaged discrete variables and therefore reflect overall inter-system agreement across experimental conditions rather than stride-by-stride waveform error. However, this method underestimates the true error, as it fails to capture within-subject variability. For each joint angle and plane of motion, the mean difference between the IMU and OMC systems was first computed at each level of walking speed (slow, normal, fast) and time (0, 5, and 10 min). RMSE was then calculated across these condition-specific mean differences as:

$$RMSE=\sqrt{\frac{1}{6}\times \sum_{i=1}^{6}{\left(\theta_{Vicon\;OMC,i}-\;\theta_{Noraxon\;IMU,i}\right)}^{2}}$$

where $\theta_{Vicon\;OMC,i}$ and $\theta_{Noraxon\;IMU,i}$ represent the condition-averaged joint angle values obtained from Vicon OMC and Noraxon Ultium™ IMU systems at six levels of different time and speed conditions.

In addition to discrete and agreement analyses, Statistical Parametric Mapping (SPM1D) was employed to assess continuous waveform-level differences between the two systems for each joint angle across the full gait cycle (0–100%). A paired SPM{t} test was applied to compare the time-normalized joint angles (pelvis 3D, hip 3D, knee, and ankle) between Vicon OMC and Noraxon Ultium™ IMU. The SPM critical threshold (α = 0.05) was computed based on random field theory, and temporal regions of significant differences were identified when the t-statistic exceeded the critical threshold. All SPM analyses were performed using MATLAB SPM1D [28,29].

## 3. Results

### 3.1. Discrete Kinematics (Max, Min, ROM) with Speed & Time Factors Between Two Systems

#### 3.1.1. 3-Factors Repeated ANOVA

A summary of all ANOVA results is presented in Table A1. Overall, walking speed was the dominant factor influencing joint kinematics, with consistent effects observed across multiple joints and planes. Device effects were present between the Noraxon Ultium™ IMU and Vicon OMC systems, with differences varying by planes and joint angles. In contrast, time effects were limited, indicating minimal influence of measurement duration on kinematic outcomes.

##### Sagittal Plane

In the sagittal plane, strong and consistent effects of walking speed were observed across all joints. Hip and knee kinematics showed significant speed-dependent increases in Max and ROM (hip flexion: *p* = 0.003; hip extension: *p* = 0.002; hip ROM: *p* < 0.001; knee flexion and ROM: *p* < 0.001), reflecting expected biomechanical modulation with faster walking.

Device effects were limited, but Device × Speed interactions were found significant for pelvic tilt (*p* = 0.018) and knee flexion peak and ROM (*p* = 0.035 and *p* < 0.01), indicating that both systems captured speed-related changes but differed in the magnitude of these responses.

Time effects were generally small, although modest influences were observed in knee kinematics (peak flexion: *p* = 0.021; ROM: *p* = 0.018). At the ankle, significant device effects were evident, with the IMU showing a consistent offset (~5°) relative to OMC for both dorsiflexion and plantarflexion (*p* ≤ 0.001).

##### Frontal Plane

In the frontal plane, walking speed significantly influenced joint kinematics, affecting pelvic obliquity (max: *p* = 0.008; min: *p* = 0.016; ROM: *p* < 0.001) and hip adduction/abduction (*p* = 0.034; ROM: *p* < 0.001).

Device effects were more present than in the sagittal plane, particularly for pelvic elevation list (*p* = 0.004) and hip abduction (*p* = 0.001), indicating systematic inter-system differences in frontal-plane measurements. A significant Device × Speed interaction was also observed significant for hip abduction (*p* = 0.023).

Time effects remained limited but were present for select variables, including minimum pelvic obliquity (*p* = 0.035) and hip ROM (*p* = 0.005). Additional interaction effects (Device × Time and Speed × Time) were observed in pelvic variables (*p* ≤ 0.03), although these were not consistent across all measures.

##### Transverse Plane

In the transverse plane, results became more variable and less consistent across joints. Walking speed significantly influenced pelvic rotation ROM (*p* = 0.006) and hip rotation (peak: *p* = 0.01; ROM: *p* = 0.001).

Device effects and interactions were more evident, particularly for hip rotation, where Device × Speed (*p* = 0.002) and Device × Time interactions (*p* = 0.028 and *p* = 0.014) were detected. Time also influenced hip rotation ROM (*p* = 0.004), although its overall impact remained secondary.

These findings indicate that both systems captured different rotational changes at varied speeds, but with greater variability and system-dependent differences compared to sagittal-plane kinematics.

#### 3.1.2. Post-Hoc—Summary

Post-hoc comparisons (Summarized in Table A2) clarified system- and speed-dependent differences in the sagittal, frontal, and transverse planes. The time effect was not found significant across joint angles in post hoc analyses.

In the sagittal plane, hip flexion was higher at faster speeds, with Vicon OMC detecting increases between normal and fast walking (*p* = 0.02), whereas Noraxon Utium™ IMU primarily detected differences between slow and fast walking (*p* = 0.034). Hip extension increased at higher speeds, with OMC showing differences across both normal–slow and slow–fast comparisons (*p* = 0.028–0.036), but IMU detecting a more limited change (normal vs. slow, *p* = 0.044). Hip ROM increased with speed in both systems (*p* ≤ 0.028), indicating consistent detection of overall joint excursion.

Peak knee flexion increased at higher speeds in both systems (OMC: *p* = 0.004; IMoCAP: *p* = 0.015), but knee ROM increases were more consistently detected in OMC (*p* = 0.002) than in IMoCAP. Ankle plantarflexion became more pronounced at faster speeds in both systems (OMC: *p* = 0.021; IMoCAP: *p* = 0.018), but ankle ROM changes were only significant in OMC (*p* = 0.009).

These findings indicate that both systems capture the expected increase in sagittal-plane motion with speed, but OMC consistently detects a broader and more sensitive range of changes, while IMU identifies the primary trends but with reduced sensitivity to intermediate speed differences.

In the transverse plane, results were more system dependent. Pelvic rotation showed greater rotational magnitude at faster speeds, but this effect was primarily detected by OMC (*p* ≤ 0.045). In contrast, hip rotation changes were more evident in the IMoCAP, with increased rotation observed from slow to normal and slow to fast speeds (*p* ≤ 0.041). This indicates that both systems captured speed-related rotational changes, but not consistently in the same variables, suggesting differences in how each system detects axial motion.

Overall, post-hoc analyses demonstrated that the Noraxon Ultium™ IMU and Vicon OMC systems were comparable in detecting sagittal-plane speed-related changes, particularly at the hip and knee, but differed in sensitivity, with OMC capturing more consistent and graded changes across speeds. In contrast, transverse-plane responses were more variable and system-dependent, indicating reduced agreement for rotational kinematics.

#### 3.1.3. Device Agreement—Bland–Altman

Bland–Altman analyses revealed generally low bias ranging from approximately 1–8° across all kinematic outcomes (Summary Tables are listed in Table A3, Table A4 and Table A5).

For maximum joint angles, the bias between Vicon OMC and Noraxon Ultium™ IMU remained small across most variables. In the sagittal plane, pelvic tilt (bias = 1.01°, 95% CI: 0.65–2.46°), hip flexion/extension (2.12°, 1.48–3.22°), and knee flexion/extension (1.32°, 0.54–2.44°) showed positive biases indicating slight IMU underestimation. Ankle dorsiflexion/plantarflexion demonstrated a larger negative bias (–7°, –7.69 to –6.35°), reflecting systematic overestimation of peak angles by the IMoCAP. Frontal plane biases were small, with pelvic list (1.28°, 1.15–1.71°) showing consistent elevation and hip adduction/abduction showing mild negative bias (–1.63°, –1.86 to –0.96°). In the transverse plane, pelvic rotation had a small positive bias (1.95°, 0.96–2.38°), whereas hip rotation showed slight negative bias (–1.63°, –2.70 to –0.77°) (see Table A3).

For minimum joint angles, sagittal plane biases remained small to moderate. Pelvic tilt showed a slight negative bias (–0.75°, –0.68 to 1.58°), hip flexion/extension displayed a positive bias (2.87°, 2.00–4.27°), and knee flexion/extension showed a mild positive bias (1.32°, 0.74–2.14°). Ankle minimum angles showed a modest negative bias (–2.25°, –3.09 to –2.04°). Frontal plane variables showed consistent negative biases for pelvic list (–2.57°, –2.37 to –1.30°) and hip adduction/abduction (–4.08°, –4.70 to –3.46°), indicating repeated IMoCAP underestimation of minimum frontal-plane positions. In the transverse plane, pelvic rotation (–2.61°, –3.30 to –1.79°) and hip internal/external rotation (–3.10°, –3.28 to –0.80°) also showed systematic underestimation (see Table A4).

ROM biases were consistent with the maximum and minimum trends. In the sagittal plane, pelvic tilt (1.56°, 0.65–2.46°), hip flexion/extension (2.35°, 1.48–3.22°), knee flexion/extension (1.49°, 0.54–2.44°), and ankle motion (–4.46°, –5.13 to –3.80°) showed small to moderate biases. Frontal plane ROM showed a small positive bias for pelvic list (1.43°, 1.15–1.71°) and a moderate positive bias for hip adduction/abduction (2.87°, 2.16–3.16°). In the transverse plane, pelvic rotation exhibited a mild positive bias (1.67°, 0.96–2.38°), while hip rotation showed a negative bias (–1.73°, –2.70 to –0.77°) (see Table A5).

#### 3.1.4. Reliability—Intraclass ICC (3,1)

The Noraxon Ultium™ IMU system demonstrated generally fair to good absolute agreement to the Vicon OMC with ICC (3,1) values varying by joint, plane, and variable (Max, Min, and ROM; see Summary in Table A6). In the sagittal plane, most joint angles showed moderate reliability, including pelvis tilt (ICC = 0.66–0.73), hip flexion/extension (ICC = 0.649–0.762), knee flexion/extension (ICC = 0.70 for ROM), and ankle dorsiflexion/plantarflexion (ICC = 0.65–0.74). Only pelvis tilt ROM (ICC = 0.39) and knee flexion minimum angle (ICC = 0.46) exhibited poor reliability.

In the frontal plane, reliability diminished. Pelvis list demonstrated moderate reliability for the maximum angle (ICC = 0.73), but minimum angle and ROM were poor (ICC = 0.42–0.46). Hip adduction/abduction showed a similar pattern, with moderate reliability for the maximum angle (ICC = 0.71) and poor reliability for minimum angle and ROM (ICC = 0.44–0.46).

In the transverse plane, agreement ranged from poor to moderate. Pelvis rotation demonstrated moderate reliability for maximum angle and ROM (ICC = 0.627 and 0.666, respectively), but minimum angle reliability was poor (ICC = 0.370). Hip internal/external rotation showed the lowest reliability overall, with moderate reliability for the maximum angle (ICC = 0.549) and poor reliability for both minimum angle and ROM (ICC = 0.177–0.262).

Overall, the Noraxon IMoCAP system exhibited the highest reliability versus OMC in the sagittal plane, moderate reliability in the frontal plane, and the lowest reliability in the transverse plane. This pattern is consistent with known challenges in capturing transverse-plane rotations using wearable IMoCAPs due to magnetometer drift and soft-tissue artifact.

#### 3.1.5. RMSE

Sagittal-plane joint angles demonstrated the lowest RMSE values across the lower limb (Table A1). Pelvic tilt showed minimal error (Max = 1.1°, Min = 1.3°, ROM = 0.3°), indicating strong correspondence between the two systems. Similarly, knee flexion/extension displayed small errors for peak angles (Max, Min = 1.1°, and ROM = 1.8°). Hip flexion/extension exhibited moderate errors for peak angles (Max = 1.7°, Min = 2.2°); however, hip ROM produced a substantially higher RMSE (5.5°), indicating higher discrepancy in estimating total excursion. Ankle dorsiflexion/plantarflexion demonstrated moderate sagittal-plane errors (Max = 5.0°, Min = 1.9°, ROM = 1.3°), reflecting systematic offsets in distal joint estimation.

RMSE values in the frontal plane were moderate and relatively consistent across variables. Pelvic list demonstrated RMSE values of 1° for maximum, 1.3° for minimum, and 0.4° for ROM. Hip adduction/abduction showed slightly minimal errors (Max = 1°, Min = 2.9°, ROM = 0.6°). These results indicate moderate agreement between the two systems for frontal-plane measures.

Transverse-plane angles showed the largest RMSE values. Pelvic rotation exhibited moderate to high errors (Max = 1.2°, Min = 1.9°, ROM = 2.1°). Hip internal/external rotation demonstrated similar magnitudes, with RMSE of 1.3° for maximum rotation, 1.6° for minimum rotation, and 0.2° for ROM. 

### 3.2. Observation of Continuous Variables with Speed & Time Factors Between Two Systems

**Figure 4:**
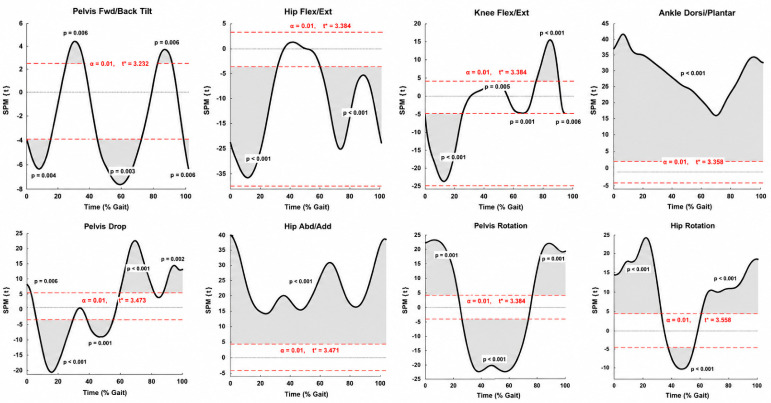
Results of the Statistical Parametric Mapping (SPM) paired *t*-tests comparing average Waveforms between Vicon OMC and Noraxon Ultium™ IMU. The waveforms were analyzed for sagittal joint angles (pelvis tilt, hip flexion/extension, knee flexion/extension, and ankle dorsiflexion/plantarflexion), frontal angles (pelvis list and hip abduction/adduction) and transverse angles (pelvis rotation and hip rotation). Note: Dashed line represents the critical threshold (α) for statistically significant. Shaded regions indicate significant differences—Šidák correction (α = 0.01) (*p* < 0.05) and non-shaded regions within limits indicate no differences between Vicon OMC and Noraxon Ultium™ IMU.

Statistical Parametric Mapping (SPM) paired *t*-tests with Šidák correction (α = 0.01) revealed significant differences between the Vicon OMC and Noraxon Ultium™ IMU systems at various instants during the gait cycle across all joint angles and planes (see Figure 4). Significant supra-threshold clusters (*p* < 0.006 to *p* < 0.001) were identified in every kinematic variable throughout the gait cycle, indicating that systematic deviations were not confined to isolated gait events but occurred across multiple phases of stance and swing.

In the sagittal plane, differences were generally phase-specific for the pelvis, hip, and knee, whereas ankle dorsiflexion/plantarflexion exhibited significant differences across nearly the entire gait cycle. Frontal-plane kinematics showed multiple significant clusters, with hip abduction/adduction demonstrating persistent waveform differences throughout the gait cycle. Transverse-plane variables exhibited the greatest disagreement, with pelvic and hip rotation displaying significant clusters across most phases of gait (see Table A6). Overall, SPM demonstrated significant waveform-level differences between the Noraxon Ultium™ IMU and Vicon OMC systems across all joint angles. These findings indicate that, although discrete variables (i.e., peak angles and ROM) demonstrated moderate agreement between systems, continuous waveforms exhibited phase-dependent differences in kinematic estimation throughout the gait cycle.

## 4. Discussion

The effectiveness of rehabilitation training increasingly relies on clinically grounded evidence derived from quantitative gait motion analysis. This study investigated the concurrent validity and reliability of an inertial measurement unit-based motion capture system (IMoCAP using Noraxon Ultium™ IMU) relative to a reference-standard laboratory-based optical motion capture system (Vicon OMC) for measuring lower-limb kinematics while walking across different speeds and time conditions. It was hypothesized that the Noraxon Ultium™ IMU system would demonstrate strong agreement with Vicon OMC in sagittal-plane kinematics, with reduced accuracy expected in the frontal and transverse planes. The results partially supported this hypothesis, demonstrating low bias (~2–4°) and moderate agreement in sagittal hip and knee kinematics, poor to moderate consistency in the frontal plane, and poor accuracy in the transverse plane, consistent with both the study hypotheses.

The three-factor ANOVA revealed significant main effects and Device × Speed interactions that clarify how each system responds to kinematic variability induced by gait velocity. In the sagittal plane, both systems captured the expected increases in hip and knee flexion/extension angles with faster walking speeds, consistent with well-established gait mechanics in which joint kinematic scales differently with walking velocity [13]. However, several significant Device × Speed interactions, specifically for pelvic tilt, knee flexion/extension, and ankle plantarflexion, indicated that although the Noraxon Ultium™ IMoCAP reliably tracked relative speed-dependent patterns, it differed from the Vicon OMC in the magnitude of these changes. The IMoCAP increasingly underestimated joint kinematics at higher speeds, likely reflecting soft-tissue artifact, and small sensor–segment alignment shifts that become amplified with greater segmental acceleration during rapid gait [30,31]. This behavior is consistent with Yin’s study assessing the fall risk at different walking speeds in a geriatric population. Yin et al. (2025) reported similar results of speed-dependent increases in error magnitude and variability, particularly for joints with larger excursions or higher angular velocities. Together, the literature findings suggest that walking speed acts as a critical factor for performing IMoCAP-based gait analysis, highlighting the importance of speed-controlled protocols and within-subject comparisons when interpreting IMoCAP-derived kinematics [25].

In the frontal and transverse planes, systematic device effects were observed across several variables, indicating consistent offsets between systems that were largely independent of walking speed. When comparing the mean differences, the Noraxon Ultium™ IMU underestimated pelvic obliquity and hip adduction by approximately 2–4° in frontal plane, while discrepancies in pelvic and hip rotation reached approximately 5–10° in transverse plane (see Table A2). These findings are consistent with previous IMoCAP validation studies reporting higher errors in non-sagittal planes due to cross-axis coupling, reduced signal-to-noise ratio, and diminished orientation stability in magnetically disturbed indoor environments [12,13,15]. The presence of speed-related differences in pelvic and hip rotation further underscores the sensitivity of IMoCAP orientation estimates to dynamic 3D motion, where soft-tissue vibration and rapid segment transitions distort inertial and magnetic signals and increase levels of uncertainty [16,18]. These findings demonstrate that while the IMoCAP system captured sagittal-plane gait modulation with reasonable accuracy, its performance in the frontal and transverse planes are subject to speed-induced error.

The Bland–Altman analysis further characterized inter-system agreement by quantifying both the magnitude and direction of measurement differences between the Noraxon Ultium™ IMU and Vicon OMC systems. Sagittal-plane biases were generally small (<3° for Max and Min while ~4° for ROM), with limits of agreement (LoA) generally less than ±5° (Table A3). These findings indicate slight IMoCAP underestimation relative to OMC (especially for Min measurement), but support moderate agreement and acceptable accuracy for sagittal-plane peak kinematics at the group level (Max and ROM measurements consistent with recent IMoCAP validation reports in [25]). Ankle dorsiflexion/plantarflexion exhibited a larger negative bias (approximately −7°), reflecting systematic IMoCAP overestimation of peak ankle angles. This behavior is consistent with the higher RMSE observed for ankle kinematics (~5°; see Table A1) and highlights the increased challenge of accurately capturing distal joint motion, where higher segment accelerations or inertial effects amplify measurement error [32,33], or the possibility of poor model calibration. In the frontal and transverse plane, joint-angle biases remained small <4°, but wider LoA up to ±11° (see Table A3, Table A4 and Table A5), reducing device agreement in measuring frontal and transverse kinematics. Together, these results demonstrate that low bias does not imply strong agreement, and that variability must be considered alongside systematic differences [16].

The RMSE findings further clarified the accuracy profile of the Noraxon Ultium™ IMU relative to the Vicon OMC and were consistent with trends commonly reported in prior IMU validation literature. In the sagittal plane, RMSE values were generally low (approximately 0.3–2.5°) (see Table A1). These errors fall well within the <5° benchmarks frequently cited for commercial IMoCAP systems during healthy gait [2,6,7,9,10,13] and align with the ANOVA results showing strong consistency between devices in capturing speed-driven changes in sagittal-plane mechanics. Hip flexion/extension ROM demonstrated a moderately elevated RMSE (~5.5°), exceeding typical peak-angle errors but remaining within ranges reported for proximal segment excursion [9,10]. Although this discrepancy may be partially influenced by subject-related variability in interpreting instructed walking speeds, it is more likely driven by the combined influence of soft-tissue artifact, pelvis–thigh coupling, and differences in anatomical versus sensor coordinate alignment, which disproportionately affect proximal segments [16,31]. Ankle maximum-angle RMSE values of 6–7° reflect the greater challenge of accurately capturing distal-segment dynamics, where higher accelerations and inertial loading amplify fusion error and integration drift [11,30].

Frontal-plane RMSE values ranged from approximately 1–3°, indicating moderate agreement between systems. These values are consistent with previously reported frontal-plane IMoCAP performance, where smaller angular excursions and increased sensitivity to sensor placement contribute to higher variability compared with sagittal-plane measures [13,15]. The IMU’s underestimation of pelvic list (obliquity) and hip abduction/adduction observed in the ANOVA is reflected in these moderate RMSE magnitudes and highlights the challenge of isolating low-amplitude frontal-plane movements using wearable sensors.

Transverse-plane RMSE values were higher relative to sagittal and frontal measures, particularly for pelvic rotation ROM (~2–3°), consistent with literature describing reduced accuracy for axial rotations in magnetically disturbed environments [12,18]. These findings correspond with the significant Device × Speed interactions observed for pelvic and hip rotation and indicate that dynamic, multi-planar motion further degrades IMoCAP heading accuracy. Because transverse-plane estimation depends heavily on magnetometer stability and sensor fusion algorithms’ ability to separate axial rotation from concurrent sagittal and frontal motion [13], reduced accuracy in this plane is expected and consistent with prior validation studies [7,9,12,30].

Intraclass reliability analysis (ICC) demonstrated generally moderate consistency between the IMoCAP and OMC systems for sagittal-plane variables, with most ICC values ranging from 0.65 to 0.74 (Table A7). Hip flexion/extension, knee flexion/extension, and ankle dorsiflexion/plantarflexion exhibited moderate reliability for peak angles and ROM, indicating stable relative ranking between systems despite systematic offsets in absolute magnitude. Pelvic tilt demonstrated moderate reliability for peak angles, whereas ROM exhibited reduced reliability, and knee flexion minimum showed poor reliability, likely reflecting increased variability near terminal stance where joint excursions are small and more susceptible to noise. Frontal-plane reliability was more variable, with moderate ICC values for maximum pelvic obliquity (ICC = 0.727) and hip adduction/abduction (0.709) but only poor reliability for minimum angles and ROM (ICC = 0.421–0.459) (see Table A7). Transverse-plane reliability was the lowest overall, particularly for hip internal/external rotation minimum and ROM, which demonstrated poor ICC values. Thus, while transverse-plane biases were small on average, poor reliability, and wide limits of agreement underscore limited consistency in axial rotation estimation.

**Figure 5:**
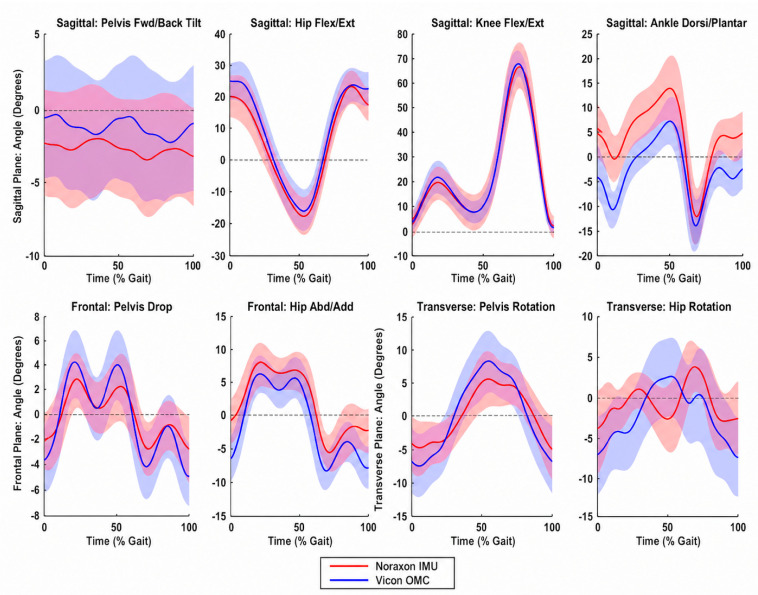
Plot of average joint angles concurrently recorded between Vicon OMC and Noraxon Ultium™ IMoCAP. The joint angle channels were grouped into cardinal planes for further investigation (Sagittal plane includes pelvis tilt, hip flexion/extension, knee flexion/extension, and ankle dorsiflexion/plantarflexion; frontal plane includes pelvis drop and hip adduction/abduction; and transverse plane includes pelvis rotation and hip internal/external rotation).

Finally, pairwise *t*-test SPM (*p* < 0.05) analyses revealed significant waveform-level differences between systems across all joints and planes. In the sagittal plane, discrepancies were concentrated during early stance and terminal swing phases characterized by high angular acceleration and intersegmental coupling. The ankle exhibited differences throughout the gait cycle, suggesting persistent offset (~5–7°, especially maximum in the ankle angle) probably caused by different methods of model calibration. The model calibration in the Vicon OMC relied on static standing capture while the Noraxon Ultium™ IMU relied on the on-board calibration obtained by the walking trial. In the frontal and transverse planes, near-continuous waveform discrepancies indicated systematic orientation biases (see Figure 5), likely arising from magnetometer misalignment and soft-tissue motion [12,21]. Although Bland–Altman analysis, RMSE, and ICC demonstrated moderate agreement for several discrete sagittal-plane variables, the SPM findings indicate that the Noraxon Ultium™ IMU and Vicon OMC systems cannot be considered fully interchangeable when evaluating continuous kinematic waveforms. An additional factor that may have contributed to the observed inter-system differences is the use of different calibration strategies. The Vicon OMC workflow established anatomical coordinate systems through a static calibration using anatomical landmarks, whereas the Noraxon Ultium™ IMU system employed a functional walking calibration to initialize sensor orientation relative to the body segments. Berner et al., 2020 reported that repeated calibration substantially influenced within-session reliability and highlighted the importance of achieving consistent sensor-to-segment alignment before inverse kinematics calculations [9]. Likewise, Rekant et al., 2022 demonstrated that calibration strategy and biomechanical modeling contribute substantially to waveform-level disagreement even when discrete kinematic variables exhibit reasonable agreement [33]. These findings indicate that agreement in peak angles and ROM does not necessarily imply equivalence throughout the entire gait cycle. While Bland–Altman analysis, RMSE, and ICC demonstrated moderate agreement for many discrete sagittal-plane variables, SPM identified persistent phase-specific deviations during weight acceptance, single-limb support, and propulsion. In general, the Noraxon Ultium™ IMU and Vicon OMC systems cannot be considered interchangeable at the waveform level because its functional calibration might contribute to early systematic offsets in the gait cycle.

Overall consideration of Bland–Altman, RMSE, and ICC results provides important insight into the nature of IMoCAP-derived measurement error. In both the present study and Yin et al. (2025), low RMSE and moderate ICC values in the sagittal plane (typically measured joint flexion/extension) support the validity of IMoCAPs for flexion–extension kinematics. Given the relatively low RMSE values and moderate agreement observed in this plane, the Noraxon Ultium™ IMU appears suitable for monitoring these clinically relevant outcomes [25]. The larger variability observed in frontal-plane measurements may therefore limit the sensitivity of the IMU system for detecting subtle biomechanical changes or asymmetries (i.e., pelvic stability, hip abductor function, and knee loading patterns). The limitations were even more pronounced in the transverse plane (i.e., often measured foot progression angle, joint rotational angle), where lower reliability and persistent waveform deviations were observed. Therefore, caution is warranted when using IMU-derived rotational measures for clinical decision-making or detailed biomechanical investigations.

For practical applications, the IMoCAP’s portability, low cost, and rapid setup offer advantages in environments lacking optical systems, if sensor alignment and calibration procedures are carefully maintained. In particular, the Noraxon Ultium™ IMU may be more appropriately applied for monitoring general gait trends, rehabilitation progression, or within-subject longitudinal changes under controlled conditions, where relative biomechanical changes may be more clinically meaningful than exact kinematic equivalence. Applications requiring precise multi-planar joint quantification or detailed pathological gait interpretation should continue to rely on laboratory-based motion capture systems.

## 5. Limitation and Future Study

A critical consideration in interpreting the present findings is the role of model calibration and biomechanical modeling in determining IMoCAP-derived joint kinematics. As emphasized by Berner et al. (2020), the accuracy of IMoCAP-based motion capture depends strongly on the calibration procedure used to align sensor coordinate systems with anatomical reference frames [9]. Small deviations during static calibration, sensor placement, or segment alignment can introduce systematic offsets that persist across all movement conditions and directly influence the IK solutions.

These calibration-dependent effects are further compounded by modeling assumptions, including joint constraint definitions, segment scaling, and coordinate system conventions, all of which can alter joint angle estimation. As discussed in Rekant et al. (2022), discrepancies between IMoCAP and optical motion capture systems should not be attributed solely to sensor limitations, but rather to the combined influence of sensor characteristics, calibration quality, and computational model [33]. In the present study, both the Noraxon Ultium™ IMU and Vicon OMC systems relied on their own modeling workflows described in Section 2.4 (kinematic modeling), which likely contributed to the observed inter-system differences, particularly in the frontal and transverse planes. In summary, the observed differences between systems in this study likely reflect the combined effects of hardware performance, calibration procedures, joint coordinate definitions, inverse kinematics constraints, and model-specific assumptions rather than isolated IMU sensor error alone.

This study evaluated healthy adults in a controlled laboratory environment, representing near ideal conditions for IMoCAP operation. However, the broader objective is to support the application of wearable IMoCAP systems. Furthermore, real-world and field-based gait environments may introduce greater magnetic disturbances and environmental noise, which could further affect IMoCAP orientation estimates.

Another limitation of this study is the relatively modest sample size (N = 10), which may have reduced statistical sensitivity for detecting smaller main effects and higher-order interactions. Although the sample size was comparable to previous IMU validation studies conducted under controlled laboratory conditions, larger cohorts would improve the generalizability of the findings [7,9]. To evaluate the robustness of the significant speed x device effects observed in the present study, post-hoc power analyses were conducted using G*Power (version 3.1.9.7) based on the observed effect sizes (derived from Cohen’s d value from post-hoc pairwise across all speed x device comparisons). The result demonstrated moderate statistical power in sagittal plane and low statistical power in frontal and transverse plane (refer to Table A2), suggesting that the study design was not sufficiently sensitive to detect meaningful speed-dependent differences between systems. The observed power mostly improves in the main effect of devices in this study.

Future research should therefore extend this validation framework to individuals with lower-limb gait complications (i.e., lower limb amputation, cerebral palsy, stroke, and OA) and to ambulatory settings outside the laboratory. When expanding validation to populations with atypical gait, an increased sample size is strongly recommended to improve statistical power and generalizability; prior work by Yin et al. (2025), which investigated IMoCAP-based gait analysis in a geriatric cohort (N = 40), suggests that sample sizes in the range of 20–40 participants are appropriate for capturing population-level variability in clinical gait studies [25].

## 6. Conclusions

Overall, the statistical and reliability analyses demonstrated that walking speed was the primary factor influencing lower-limb kinematics across both systems, whereas time had minimal effects. Agreement between the Noraxon Ultium™ IMU and Vicon OMC was strongest in the sagittal plane, moderate in the frontal plane, and weakest in the transverse plane. In general, wearable technology such as Noraxon Ultium™ IMU systems can offer a practical and controlled sagittal-plane gait assessment in healthy individuals potentially applying for evaluating clinical gait analysis for lower limb diseases and complications, particularly in settings where optical motion capture is impractical. These findings should be interpreted as preliminary methodological validation results and should not yet be generalized to pathological gait populations without further investigation.

## Data Availability

Dataset available on request from the authors. The raw data supporting the conclusions of this article will be made available by the authors on request.

## Abbreviations

The following abbreviations are used in this manuscript:

IMoCAP	IMU (Inertial Measurement Unit) motion capture
OMC	Optical motion capture
RMSE	Root Mean Square Error
SPM	Statistical Parametric Mapping
LoA	Limit of Agreement
CI	Confident Interval
ICC	Intraclass Reliability

## Appendix A

**Table A1:** Summary of three-factor ANOVA and interaction effects for all kinematic parameters.

Joint Angle (Deg)	Variable	System	Mean	SD	RMSE	Interaction Effects (*p* < 0.05)					
						Speed	Time	Device × Speed	Device × Time	Speed × Time	Device × Speed × Time
Pelvis Tilt	Max	OMC	0.9	5.0	1.1	0.094	0.828	0.018 *	0.749	0.651	0.818
		IMoCAP	−0.6	3.9							
	Min	OMC	−3.1	4.8	1.3	0.085	0.654	0.687	0.947	0.584	0.767
		IMoCAP	−4.9	3.8							
	ROM	OMC	4.4	1.9	0.3	0.002 *	0.288	0.455	0.043 *	0.294	0.507
		IMoCAP	4.2	1.4							
Hip Flexion /Extension	Max	OMC	26.9	5.5	1.7	0.003 **	0.5	0.098	0.104	0.132	0.319
		IMoCAP	24.6	4.6							
	Min	OMC	−15.2	7.6	2.2	0.002 **	0.32	0.186 *	0.406	0.482	0.55
		IMoCAP	−18.3	4.8							
	ROM	OMC	42.0	4.2	5.5	<0.001 **	0.121	0.063	0.306	0.022 *	0.376
		IMoCAP	43.7	5.6							
Knee Flexion /Extension	Max	OMC	69.5	4.7	1.1	<0.001 **	0.021 *	0.035 *	0.558	0.056	0.457
		IMoCAP	68.0	6.8							
	Min	OMC	2.5	1.7	1.1	0.341	0.774	0.117	0.985	0.617	0.215
		IMoCAP	1.1	4.0							
	ROM	OMC	66.3	4.9	1.8	<0.001 **	0.018 *	<0.01 **	0.239	0.093	0.04 *
		IMoCAP	67.6	4.3							
Ankle Dorsiflexion /Plantarflexion	Max	OMC	8.5	3.7	5.0	0.467	0.114	0.945	0.401	0.367	0.616
		IMoCAP	15.5	4.5							
	Min	OMC	−15.4	4.3	1.9	0.003 **	0.264	0.068	0.709	0.048 *	0.284
		IMoCAP	−12.8	4.2							
	ROM	OMC	28.2	4.8	1.3	<0.001 **	0.089	0.058	0.436	0.08	0.28
		IMoCAP	28.5	7.0							
Pelvis List (Obliquity)	Max	OMC	4.6	1.9	1.0	0.008 *	0.267	0.588	0.009 **	0.9	0.775
		IMoCAP	3.2	2.0							
	Min	OMC	−6.3	2.2	1.3	0.016 *	0.035 *	0.841	0.655	0.516	0.068
		IMoCAP	−4.5	1.9							
	ROM	OMC	7.9	3.2	0.4	<0.001 **	0.122	0.425	0.103	0.03 *	0.182
		IMoCAP	7.4	2.4							
Hip Adduction /Abduction	Max	OMC	6.2	2.3	1.0	0.442	0.45	0.508	0.391	0.468	0.595
		IMoCAP	7.6	3.2							
	Min	OMC	−10.1	2.0	2.9	0.034 *	0.143	0.023 *	0.889	0.521	0.429
		IMoCAP	−6.1	2.9							
	ROM	OMC	14.1	2.5	0.6	<0.001 *	0.005 *	0.458	0.831	0.199	0.128
		IMoCAP	13.2	2.6							
Pelvis Rotation	Max	OMC	8.3	5.0	1.2	0.075	0.434	0.001 **	0.146	0.827	0.246
		IMoCAP	6.6	3.4							
	Min	OMC	−8.3	3.0	1.9	0.052	0.522	0.002 *	0.7	0.651	0.541
		IMoCAP	−5.8	3.4							
	ROM	OMC	13.7	5.3	2.1	0.006 *	0.17	0.317	0.055	0.583	0.25
		IMoCAP	11.0	4.6							
Hip Internal /External Rotation	Max	OMC	3.5	5.1	1.3	0.131	0.057	0.322	0.372	0.301	0.689
		IMoCAP	5.3	2.6							
	Min	OMC	−9.9	4.1	1.6	0.01 *	0.172	0.168	0.028 *	0.021 *	0.374
		IMoCAP	−7.8	3.1							
	ROM	OMC	13.0	2.9	0.2	0.001 **	0.004 **	0.517	0.014	0.397	0.157
		IMoCAP	13.2	3.2							

Note: * = *p* < 0.05 and ** = *p* < 0.001.

**Table A2:** Summary of Post-hoc Speed * Device Interaction (*p* < 0.05) for all kinematic parameters.

Cardinal Plane	Joint Angle (Deg)	Variable	Device			Observed Power Speed × Device		Post-Hoc—Speed × Device (*p*-holm)					
								Vicon OMC			Noraxon Ultium™ IMU		
			MeD	SE	p*	ES	G*P	Norm Vs. Slow	Norm Vs. Fast	Slow Vs. Fast	Norm Vs. Slow	Norm Vs. Fast	Slow Vs. Fast
Sagittal	Pelvis Tilt	Max	1.6	1.4	0.293	0.30	0.14	1.000	0.535	1.000	1.000	0.130	1.000
		Min	1.9	1.2	0.157	0.30	0.14	1.000	0.642	1.000	1.000	1.000	1.000
		ROM	0.2	0.3	0.458	0.10	0.06	1.000	0.577	0.241	1.000	0.157	1.000
	Hip Flexion /Extension	Max	2.4	1.3	0.104	0.40	0.21	0.922	0.02	0.102	0.106	0.057	0.034
		Min	3.2	1.8	0.108	0.40	0.21	0.028	0.248	0.036	0.044	0.312	0.052
		ROM	−1.7	2.0	0.412	−0.30	0.14	0.003	0.003	0.002	0.028	0.003	0.003
	Knee Flexion /Extension	Max	1.5	1.4	0.315	0.20	0.08	0.004	0.086	0.003	0.015	0.863	0.015
		Min	1.5	1.0	0.198	0.40	0.21	1.000	1.000	1.000	1.000	1.000	1.000
		ROM	−1.3	2.3	0.586	−0.20	−0.09	0.001	0.946	0.002	0.051	1.000	1.000
	Ankle Dorsiflexion /Plantarflexion	Max	−7.0	1.0	<0.001	−1.40	0.97	1.000	0.829	1.000	1.000	1.000	1.000
		Min	−2.6	0.6	0.001	0.60	0.99	0.474	0.217	0.201	0.12	0.217	0.018
		ROM	−0.3	2.6	0.902	0.19	0.72	<0.001	0.939	0.009	0.939	1.000	1.000
Frontal	Pelvis List (Obliquity)	Max	1.4	0.4	0.004	0.60	0.40	0.155	0.229	0.190	0.190	0.155	0.099
		Min	−1.8	0.8	0.052	−0.70	0.51	0.142	0.652	0.280	0.054	0.437	0.147
		ROM	0.5	1.0	0.664	0.10	0.06	0.012	0.131	0.016	0.194	0.263	0.173
	Hip Adduction /Abduction	Max	−1.4	0.6	0.051	−0.40	0.21	1.000	1.000	1.000	1.000	0.87	0.797
		Min	−4.1	0.9	0.001	−1.40	0.97	0.18	0.262	0.134	0.348	0.399	0.348
		ROM	0.9	1.0	0.422	0.30	0.14	1.000	1.000	1.000	0.18	0.307	0.156
Transverse	Pelvis Rotation	Max	1.7	1.1	0.149	0.30	0.14	1.000	0.104	0.405	1.000	0.167	1.000
		Min	−2.6	1.1	0.047	−0.70	0.51	1.000	0.045	0.206	1.000	0.542	1.000
		ROM	2.7	2.1	0.222	0.40	0.21	1.000	0.021	1.000	1.000	0.849	0.665
	Hip Internal /External Rotation	Max	−1.7	1.3	0.211	−0.30	0.14	1.000	1.000	1.000	0.032	1.000	1.000
		Min	−2.0	1.8	0.279	−0.40	0.21	0.093	1.000	0.417	1.000	1.000	1.000
		ROM	−0.3	0.7	0.726	−0.10	0.06	0.215	1.000	0.195	0.013	1.000	0.041

Note: * = *p* < 0.05. MeD: Mean Difference, p*: p-holm, ES: Effective Size, G*P: Observed G-Power for main interaction device × speed.

**Table A3:** Summary of Bland–Altman agreement between Vicon OMC and Noraxon Ultium™ IMU for Max kinematic parameters in cardinal planes (LoA: Limit of Agreement, CI: Confidence Interval).

Maximum						
Cardinal Plane	Joint Angle	Bias (°)			−1.96 SD LoA (°)	+1.96 SD LoA (°)
		Estimate (°)	Lower 95% CI	Upper 95% CI		
Sagittal	Pelvic Tilt (°)	1.01	0.65	2.46	–6.44	8.46
	Hip Flexion/Extension (°)	2.12	1.48	3.22	–5.53	9.77
	Knee Flexion/Extension (°)	1.32	0.54	2.44	–7.06	9.70
	Ankle Dorsi/Plantarflexion (°)	–7.00	−7.70	−6.35	–12.8	−1.21
Frontal	Pelvic List (°)	1.28	1.15	1.71	–1.05	3.62
	Hip Adduction/Abduction (°)	–1.63	−1.86	−0.96	–9.55	6.30
Transverse	Pelvic Rotation (°)	1.95	0.96	2.38	–4.10	8.0
	Hip Internal/External Rotation (°)	−1.63	−2.70	−0.77	–9.55	6.30

**Table A4:** Summary of Bland–Altman agreement between Vicon OMC and Noraxon Ultium™ IMU for Min kinematic parameters in cardinal planes (LoA: Limit of Agreement, CI: Confidence Interval).

Minimum						
Cardinal Plane	Joint Angle	Bias (°)			−1.96 SD LoA (°)	+1.96 SD LoA (°)
		Estimate (°)	Lower 95% CI	Upper 95% CI		
Sagittal	Pelvic Tilt (°)	–0.75	−0.68	1.58	–8.47	6.98
	Hip Flexion/Extension (°)	2.87	2.00	4.27	−7.32	13.06
	Knee Flexion/Extension (°)	1.32	0.74	2.14	–4.90	7.53
	Ankle Dorsi/Plantarflexion (°)	–2.25	−3.09	−2.04	–6.24	1.75
Frontal	Pelvic List (°)	–1.84	−2.37	−1.30	–5.95	4.14
	Hip Adduction/Abduction (°)	–4.08	−4.70	−3.46	–8.80	2.78
Transverse	Pelvic Rotation (°)	–2.61	−3.30	−1.79	–8.35	3.12
	Hip Internal/External Rotation (°)	–3.10	−3.28	−0.80	–11.0	4.76

**Table A5:** Summary of Bland–Altman agreement between Vicon OMC and Noraxon Ultium™ IMU for ROM kinematic parameters in cardinal planes (LoA: Limit of Agreement, CI: Confidence Interval).

Range of Motion (ROM)						
Cardinal Plane	Joint Angle	Bias (°)			−1.96 SD LoA (°)	+1.96 SD LoA (°)
		Mean Difference (°)	Lower 95% CI	Upper 95% CI		
Sagittal	Pelvic Tilt (°)	1.56	0.65	2.46	–6.94	10.05
	Hip Flexion/Extension (°)	2.35	1.48	3.22	–5.79	10.49
	Knee Flexion/Extension (°)	1.49	0.54	2.44	–7.39	10.40
	Ankle Dorsi/Plantarflexion (°)	–4.46	−5.13	−3.80	–10.6	1.68
Frontal	Pelvic List (°)	1.43	1.15	1.71	−1.20	4.06
	Hip Adduction/Abduction (°)	2.87	2.16	3.16	–1.72	7.46
Transverse	Pelvic Rotation (°)	1.67	0.96	2.38	–4.97	8.32
	Hip Internal/External Rotation (°)	−1.73	−2.70	−0.77	–10.7	7.25

**Table A6:** Summary of SPM paired *t*-test result with the total range of deviations between Vicon OMC and Noraxon Utium™ IMU.

Plane	Joint Angle	Cluster Difference (Approximate %Gait)	Significant Difference in Total %Gait	Waveform Agreement Interpretation
Sagittal	Pelvis Tilt	0–15%, 25–45%, 50–75%, 80–100%	~65%	✓ Moderate agreement. ✓ Ankle joint represented an exception, showing nearly continuous bias throughout gait because of calibration issue in identifying zero.
	Hip Flexion/Extension	0–25%, 65–100%	~60%	
	Knee Flexion/Extension	0–20%, 60–75%, 80–100%	~48%	
	Ankle Dorsiflexion/Plantarflexion	0−100%	100%	
Frontal	Pelvis List	0–10%, 10–30%, 40–55%, 60–95%	~70%	✓ Poor agreement. ✓ Cluster differences occurred over large portion of the gait cycle.
	Hip Abduction/Adduction	0–100%	~95%	
Transverse	Pelvis Rotation	0–20%, 25–80%, 80–100%	~90%	✓ Poor agreement. ✓ Persistent significant waveform differences across most of the gait cycle.
	Hip Rotation	0–30%, 40–60%, 70–100%	~80%	

**Table A7:** Summary of Intraclass Correlation ICC (3,1) model between Vicon OMC and Noraxon Ultium™ IMU for all kinematic parameters in cardinal planes.

Cardinal Plane	Joint Angle (°)	Variable	Intraclass Correlation ICC	Interpretation [31]
Sagittal	Pelvis Tilt (°)	Max	0.66	Moderate
		Min	0.73	Moderate
		ROM	0.69	Moderate
	Hip Flexion /Extension (°)	Max	0.65	Moderate
		Min	0.76	Moderate
		ROM	0.67	Moderate
	Knee Flexion /Extension (°)	Max	0.71	Moderate
		Min	0.46	Poor
		ROM	0.70	Moderate
	Ankle Dorsiflexion /Plantarflexion (°)	Max	0.65	Moderate
		Min	0.68	Moderate
		ROM	0.74	Moderate
Frontal	Pelvis List (Obliquity)	Max	0.73	Moderate
		Min	0.42	Poor
		ROM	0.46	Poor
	Hip Adduction /Abduction (°)	Max	0.71	Moderate
		Min	0.44	Poor
		ROM	0.46	Poor
Transverse	Pelvis Rotation (°)	Max	0.63	Moderate
		Min	0.37	Poor
		ROM	0.67	Moderate
	Hip Internal /External Rotation (°)	Max	0.55	Moderate
		Min	0.18	Poor
		ROM	0.26	Poor
